# Automatic Global Level Set Approach for Lumbar Vertebrae CT Image Segmentation

**DOI:** 10.1155/2018/6319879

**Published:** 2018-10-08

**Authors:** Yang Li, Wei Liang, Yinlong Zhang, Jindong Tan

**Affiliations:** ^1^Key Laboratory of Networked Control Systems, Shenyang Institute of Automation, Chinese Academy of Sciences, Institutes for Robotics and Intelligent Manufacturing, Chinese Academy of Sciences, Shenyang 110016, China; ^2^University of Chinese Academy of Sciences, Beijing 100049, China; ^3^Department of Mechanical, Aerospace and Biomedical Engineering, University of Tennessee, Knoxville, TN 37996, USA

## Abstract

Vertebrae computed tomography (CT) image automatic segmentation is an essential step for Image-guided minimally invasive spine surgery. However, most of state-of-the-art methods still require human intervention due to the inherent limitations of vertebrae CT image, such as topological variation, irregular boundaries (double boundary, weak boundary), and image noise. Therefore, this paper intentionally designed an automatic global level set approach (AGLSA), which is capable of dealing with these issues for lumbar vertebrae CT image segmentation. Unlike the traditional level set methods, we firstly propose an automatically initialized level set function (AILSF) that comprises hybrid morphological filter (HMF) and Gaussian mixture model (GMM) to automatically generate a smooth initial contour which is precisely adjacent to the object boundary. Secondly, a regularized level set formulation is introduced to overcome the weak boundary leaking problem, which utilizes the region correlation of histograms inside and outside the level set contour as a global term. Ultimately, a gradient vector flow (GVF) based edge-stopping function is employed to guarantee a fast convergence rate of the level set evolution and to avoid level set function oversegmentation at the same time. Our proposed approach has been tested on 115 vertebrae CT volumes of various patients. Quantitative comparisons validate that our proposed AGLSA is more accurate in segmenting lumbar vertebrae CT images with irregular boundaries and more robust to various levels of salt-and-pepper noise.

## 1. Introduction

Image-guided minimally invasive spine surgery (IG-MISS) was wildly performed for the degenerated lumbar spine in the last decades [1]. The public demand has been increased for these procedures due to its various benefits, such as performing more accurate instrumentation placement with less radiation exposure, yielding less postoperative complications, and reducing recovery time [2, 3]. In Image-guided-surgery (IGS) system, image segmentation of the interesting anatomical structure is an essential preprocessing step for 3D reconstruction and image registration, which is commonly applied in preoperative planning, intraoperative navigation, and postoperative assessment [4]. However, manually segmenting lumbar vertebrae is a time-consuming, subjective, and nonrepeatable task. Implementing an automatic lumbar vertebrae segmentation approach is of great importance not only for simplifying surgery process but also for improving operations accuracy.

**Figure 1:**
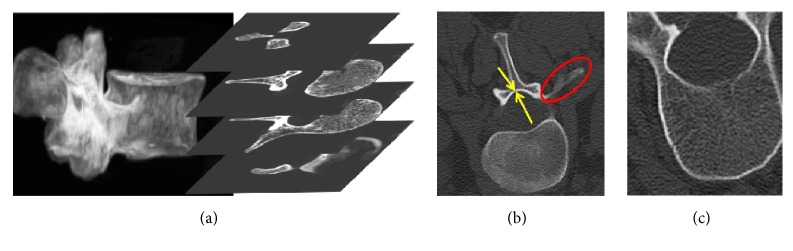
Challenges in segmentation of lumbar vertebrae CT images: (a) topological variation of vertebrae anatomical structure, (b) irregular boundaries (double boundary and weak boundary), (c) image noise.

Although a large amount of literature has been focused on lumbar vertebrae CT image segmentation, there still exist some potential challenges due to the inherent limitations of CT imaging modality and complexity of vertebrae anatomical structure.  Figure 1 shows some specific challenges on lumbar vertebrae CT image segmentation, such as topological variation of vertebrae anatomical structure, irregular boundaries (double boundary, weak boundary) [5], and image noise. Generally speaking, two major types of approaches are exploited to deal with these issues, i.e., statistical shape models and level set models. Statistical shape models (SSM) [6–11] characterized by matching a prior template to new images is able to address the problem of irregular boundaries in lumbar vertebrae CT image segmentation. Nevertheless, these models do not explicitly cope with the individual differences of the vertebrae anatomical structure, since the prior templates are mainly established by statistical means of training shapes. Additionally, these statistical methods are computationally expensive because it is heavily dependent on spatial registration of the deformable model [12]. By comparison, level set methods (LSM) [13–24] are mainly based on edge or region information, which can naturally figure out the topological variation issue. However, the edge-based LSM are mostly quite sensitive to image noise and often suffer from serious boundary leaking problems when the objects have weak boundaries [25]. On the other hand, the region-based LSM assume homogeneity of local region intensities, which cannot segment images with inhomogeneity [26]. Furthermore, the manual interaction of initial contour is still required in traditional LSM and pixels far away from the initial contour are meaningless for obtaining the object boundary [27].

Due to these limitations in CT image segmentation, a novel lumbar vertebrae CT image segmentation approach is proposed to achieve fast, robust, and accurate segmentation. Our segmentation strategy is an automatic global level set approach (AGLSA), which comprises two stages within the coarse-to-fine framework. First, we introduce an automatically initialized level set function (AILSF) which exploits hybrid morphological filter (HMF) and Gaussian mixture model (GMM) to automatically generate a smooth initial contour which is precisely adjacent to the object boundary. Second, a regularized level set formulation is designed to overcome the weak boundary leaking problem, which utilizes the region correlation of histograms inside and outside the level set contour as a global term. Ultimately, a gradient vector flow (GVF) based edge-stopping function is employed to guarantee a fast convergence rate of the level set evolution and to avoid level set function oversegmentation at the same time. Our proposed approach has been tested on 115 vertebrae CT volumes of various patients. Quantitative comparisons validate that our proposed AGLSA is more accurate in segmenting lumbar vertebrae CT images with irregular boundaries and more robust to various levels of salt-and-pepper noise. The rest of this paper is organized as follows.  Section 2 reviews the related literature.  Section 3 presents the conception of our proposed method. The evaluation methods of these approaches are then introduced, and experimental results and analysis are detailed in Section 4.  Section 5 concludes this paper.

## 2. Related Work

Lumbar vertebrae segmentation methods can be briefly classified into two types: (1) statistical shape models [12] which take shape prior information into consideration and (2) active contour models (ACM) [28] which directly take intensity information into account. SSM generate mean shapes using their own shape parameters, such as Fourier and wavelet descriptors, and use shape constraints to overcome ambiguous boundary information [6]. For instance, profound prior knowledge, such as various kinds of models covering shape, gradient, and appearance information, was utilized by Klinder et al. [7] to obtain a robust vertebrae segmentation framework. Ma et al. [8] proposed a coarse-to-fine deformable surface model based on learned bone-structure edge detectors to segment vertebrae in 3D CT images. Manifold embeddings [9] were introduced to treat multiple vertebrae as a whole shape for spine segmentation. Recently, Rasoulian and Suzani et al. [10, 11] developed a statistical multi-vertebrae shape+pose model and employed a registration-based technique to segment the CT and MR images of spine. Unfortunately, all these methods for lumber vertebrae segmentation are semiautomated requiring manually marked initial locations of vertebras and suffer from expensive computational complexity since these algorithms rely heavily on spatial registration of the deformable model. Additionally, these deformable spine models depend upon sufficiently large training and testing dataset, which increases the complexity, and these models fail to segment the vertebrae regions that are apparently distinct from the dataset images. The level set method [29], which belongs to ACM, has been widely utilized in image segmentation for its intrinsic property in dealing with topological variation. The basic idea of LSM is to evolve the zero-level of the level set function (LSF) in the image domain until it reaches the boundaries of the regions of interest [13]. LSM is able to handle the issue that topology of the contours merges and breaks, which wildly exists in the segmentation of lumbar vertebrae in CT images. LSM for image segmentation can be briefly categorized into three types: (1) edge-based models, such as distance regularized level set evolution (DRLSE) [14], use edge-detecting function to stop evolving curves which results in leaking out the ideal contours when the edges are ambiguous; Khadidos et al. [30] calculated a weighted energy term according to the relative importance of boundary points to solve the problem of weak edge leaking; (2) region-based models, such as Chan-Vese model (C-V) [15], assume object and background intensity to be homogeneous which cannot tackle the problems of intensity inhomogeneity; (3) gradient vector flow models (GVF) [16] use GVF as the external force field to extend the capture range, but they fail to efficiently solve the convergence problem for an image with deep concavities boundary and high noise level. Liu et al. [31] attempted to distinguish noises and object edge points by using the local regional properties of images points. All the methods mentioned above need manually initialized contour and the corresponding segmentation performance is sensitively dependent on it. Several researchers have come up with discrepant methods to tackle this initialization problem. For instance, Aslan et al. [5, 17, 18] have integrated intensity, spatial interaction, and shape information into a probabilistic energy model in order to obtain the optimum segmentation. Shalaby et al. [19, 20] have used a two-dimensional principal component analysis to extract the shape prior information in order to initialize level set function and constructed a probabilistic shape-based model. Lim et al. [21] have introduced an edge-mounted Willmore flow as well as a prior shape kernel density estimator, to the level set segmentation framework, but it is still in desperate need of sufficiently large training and testing dataset. Symmetry property of target boundary has been utilized by Liu et al. [22] to initialize the level set function, but the application is limited to segment symmetrical objects. A level set algorithm has been proposed by Li et al. [23], which tries to evolve level set function from the initial segmentation via spatial fuzzy clustering, yet the initial contour is not smooth enough. A simple initialization method for the level set function is developed by Huang et al. [24], which exploited Otsu method to automatically initialize LSF, but it assumed that the intensity of the regions inside and outside the object boundary was homogeneous which is incapable of conforming to the feature of lumbar vertebrae CT images. Recently, Balla-Arabe et al. [32] and Liu et al. [33] have taken advantage of 2D histogram information to constrain the level set evolution, in order to reduce the computational complexity of the LSM. All the methods above fail to consider the relevance between regions that lie inside and outside the evolving contour. In our previous work [34], a region-correlation-based LSM is designed to address this problem.

## 3. Methodology

**Figure 2:**
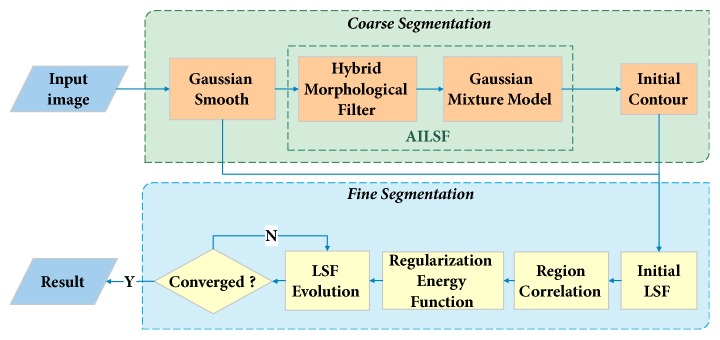
Flowchart of AGLSA.

This section details the conception of our proposed AGLSA for lumbar vertebrae CT image segmentation. The process of this approach is illustrated in Figure 2. It can be observed from the figure that AGLSA includes two stages within the coarse-to-fine framework: In the first stage, we introduce an automatically initialized level set function (AILSF) which exploits hybrid morphological filter (HMF) and Gaussian mixture model (GMM) to automatically generate a smooth and well-defined initial contour, which is precisely adjacent to the object boundary. In the second stage, a regularized level set formulation based on the region correlation of histograms inside and outside the level set contour is employed to overcome the weak boundary leaking problem and eventually to obtain the desirable segmentation.

### 3.1. Automatically Initialized Level Set Function

#### 3.1.1. Hybrid Morphological Filter

To address the salt-and-pepper noise problem, a sequence of morphological filters is performed for lumbar vertebrae CT image denoising. Let *I* be an input image, *I*(*x*, *y*) denote the gray level at pixel (*x*, *y*), and *b* denote the structuring element. Unlike binary image morphological operations [35], the erosion and dilation operators in gray images [36] are defined as follows: The erosion of *I* at pixel (*x*, *y*) with a structuring element *b* is the minimum value of the image in the region coincident with *b* when the origin of *b* is at pixel (*x*, *y*); the dilation of *I* at pixel (*x*, *y*) with a structuring element *b* is the maximum value of the image over the window *b* when the origin of *b* is at pixel (*x*, *y*). Therefore, the erosion of image *I* at pixel (*x*, *y*) is given by

(1) IΘbx,y=minIx+s,y+t−bs,t;s,t∈Db,x+s,y+t∈DI

and the dilation of image *I* at pixel (*x*, *y*) is given by

(2) I⊕bx,y=maxIx−s,y−t+bs,t;s,t∈Db,x−s,y−t∈DI.

The morphological opening operator *α* and closing operator *β* for gray images are defined as

(3) αI,b=IΘb⊕b

(4) βI,b=I⊕bΘb

where Θ and ⊕ denote erosion and dilation, respectively. Opening operator removes small objects from the foreground (usually taken as the dark pixels) of an image, while closing operator removes small holes in the background (usually taken as the bright pixels). Therefore, we introduce a HMF defined as

(5) HMFcooc,bn=βα⋯βααβI,b,b,b,b⋯bn,bn

where *n* ∈ [1, +*∞* is the number of iterations, which can control the smoothing effect of the filter. This HMF can remove the dark pixels from vertebrae regions and the bright pixels from background through several iterations of opening and closing operators, respectively. Noticeably, the reason why we take the closing operator firstly in the sequence of morphological filter is that this type of operation order is able to preserve the weak boundary.

#### 3.1.2. Gaussian Mixture Model

To automatically attain a smooth and well-defined contour for the anatomical object, the input images should be preliminarily segmented into foreground and background areas. In this regard, we take advantage of GMM to cluster the input image into two classes: background class and foreground class. GMM is characteristic of a weighted sum of *K* component Gaussian densities defined as

(6) $$\begin{array}{c} p\left(\;x\;|\;\Phi \;\right)=\overset{K}{\underset{i=1}{\sum }}\omega_{i}G\left(\;x\;|\;u_{i},\theta_{i}\;\right) \end{array}$$

where *x* is the gray value of the input image, *ω*_*i*_, *i* = 1,…, *K*, represent the mixture weights, and *G*(*x*∣*u*_*i*_, *θ*_*i*_) denotes the *i*_*th*_ component Gaussian densities with mean *u*_*i*_ and standard variation *θ*. Φ = {*ω*_1_,…, *ω*_*K*_, *θ*_1_,…, *θ*_*K*_} refers to the complete set of parameters for GMM. This set of parameters in Φ is estimated using expectation-maximization (EM) algorithm.

### 3.2. Global Level Set Approach

A global level set approach is utilized to obtain the ultimate refined lumbar vertebrae CT image segmentation result, which has been automatically initialized by the method mentioned in section A. The LSM evolves a high-dimensional surface *ϕ*(*x*, *y*, *t*) with an evolution function defined as

(7) $$\begin{array}{c} \frac{\partial \phi }{\partial t}=F\left|\;\nabla \phi \;\right| \end{array}$$

where *F* is the speed function that controls the evolution of the LSF. The main idea of edge-based LSM is to utilize gradient information to evolve the initial contour to the object boundary. The popularly used formulation of edge-based LSM is [14]

(8) ∂ϕ∂t=μ div1−1∇ϕ∇ϕ+λδεϕdivg∇I∇ϕ∇ϕ+αg∇Iδεϕ

where *g*(∇*I*) denotes the edge indicator function, *δ*_*ε*_(*ϕ*) denotes the Dirac function, and *μ*, *λ*, *α* are positive constants that control the contributions of these function evolving terms. Region-based LSM separately consider the statistical information of the entire inside and outside the contour. The classic formulation of region-based LSM is [15]

(9) ∂ϕ∂t=δεϕμ div∇ϕ∇ϕ−λ1I−c12+λ2I−c22

where *μ*, *λ*_1_, *λ*_2_ are positive constants and are the average intensities of the region inside and outside the contour, respectively. Unfortunately, these methods fail to consider the relevance between regions that lie inside and outside the contour. In this section, a global level set approach based on region correlation is designed to cope with this issue.

#### 3.2.1. Region-Correlation-Based Energy Function

Due to the presence of intensity inhomogeneity and noise, the gray values are neither constant nor continuous variation in lumbar vertebrae CT images. Thus, the average intensity values in (9) are not capable of segmenting images with these problems. To tackle this challenge, we modify (8) by considering the region correlation between regions inside and outside the contour as a global term. For a LSF *ϕ* : *Ω* ∈ *ℜ*, a novel energy function E(*ϕ*) is defined by

(10) $$\begin{array}{c} E\left(\;\phi \;\right)=\mu E_{int}\left(\;\phi \;\right)+\lambda R\left(\;\phi \;\right) \end{array}$$

where *μ*, *λ* are positive parameters that regulate the impact of energy terms. The first internal energy term *E*_int_(*ϕ*) is designed to avoid LSF from unnecessary reinitialization. It is based on the formulation proposed by [14] given by

(11) $$\begin{array}{c} E_{int}\left(\;\phi \;\right)=\int_{\Omega }p\left(\;\left|\;\nabla \phi \;\right|\;\right)dx=\frac{1}{2}\int_{\Omega }{\left(\;\left|\;\nabla \phi -1\;\right|\;\right)}^{2}dx. \end{array}$$

The second global term *R*(*ϕ*) is the external energy depending upon the region correlation between regions inside and outside the contour. We define this term as

(12a) Hinside=hijL×2,

(12b) Rϕ=∫Ωg∇IHεϕ·exp⁡MDHinside,Houtsideηdx.

Two matrices in (12a) denote the normalized region histograms inside and outside the contour, respectively; *L* denotes the gray level of the images. In (12b), *H*_*ε*_(*ϕ*) represents the Heaviside function and *η* is a positive parameter. To take the histogram information of weak boundary into consideration, we calculate the Mahalanobis distance between *H*_*inside*_ and *H*_*outside*_ as follows:

(13) MDHinside,Houtside=Hinside−HoutsideTΣ−1Hinside−Houtside

where Σ denotes the covariance matrix.

Eventually, given (10), (11), (12a), (12b), and (13), the energy functional of our method in (10) is as follows

(14) Eϕ=μ∫Ωp∇ϕdx+λ∫Ωg∇IHεϕ·expMDHinside,Houtsideηdx

which can be minimized by solving the following gradient flow:

(15) ∂ϕ∂t=μ div1−1∇ϕ∇ϕ+λg∇IδεϕexpMDHinside,Houtsideη

where *δ*_*ε*_(*ϕ*) is the Dirac delta function.

As defined above, the proposed energy function should be effective when segmenting lumbar vertebrae CT images with weak boundary in noisy conditions. This property will be demonstrated subjectively and objectively by experiments on clinical lumbar vertebrae CT images.

#### 3.2.2. Edge-Stopping Function

As mentioned above, the proposed LSM is automatically initialized by AILSF and the initial contour is already in proximity to the object boundary. Therefore, the edge-stopping function *g*(∇*I*) should quickly converge and effectively avoid LSF oversegmentation which results in weak boundary leaking. Usually, an edge-stopping function is defined as

(16) $$\begin{array}{c} g\left(\;\nabla I\;\right)=\frac{1}{1+{\left|\nabla G_{\sigma }\cdot I\right|}^{m}} \end{array}$$

where *G*_*σ*_ represents a Gaussian kernel with a standard deviation *σ* and *m* = 1,2. The edge map of gradient vector flow [37] is adopted to accelerate the convergence of *g*(∇*I*) and (17) can be rewritten as

(17) $$\begin{array}{c} g\left(\;\nabla I\;\right)=exp-{\left(\;\frac{\left|\nabla G_{\sigma }\cdot I\right|}{r}\;\right)}^{m} \end{array}$$

where *m* ∈ [2, +*∞*, *r* is a scalar that controls the extent of edge-stopping function convergence rate. Since the initial contour is already close to the boundary of lumbar vertebrae in the first stage of our approach, the exponential form of *g*(∇*I*) can make the evolution of LSF converge rapidly before it crosses the weak boundary.

## 4. Experimental Results and Analysis

### 4.1. Data

Clinical datasets provided by Microsoft Research [38] and SpineWeb [39] are employed to test the performance of our approach. The datasets consist of spine CT image scans (pixel resolution: 512 × 512) obtained from 115 different patients aging between 23 and 86 years old and these scans have 150 to 200 slices per patient. The ground-truth images are manually and accurately segmented via expert using TurtleSeg [40] open software. Our segmentation approach is implemented on Matlab R2017a platform installed on PC with 2 Intel Xeon (R) 3.07GHz CPUs, 12GB RAM, and NVIDIA Quadro 5000 graphic processor. The following parameters are determined empirically for our proposed AGLSA in all the experiments: *n* = 1, *μ* = 1.0, *λ* = 3.0, *σ* = 0.7, *η* = 2.0, *m* = 2, *r* = 0.9.

### 4.2. Evaluation Criterion

The evaluation of our segmentation approach and the corresponding comparisons with the prevailing state-of-the-art methods are conducted by means of six criteria: (1) dice similarity coefficient (DSC); (2) misclassification rate (MCR); (3) mean absolute distance (MAD); (4) Hausdorff distance (HD); (5) running time (RT); (6) iteration number. The DSC is formulated as

(18) $$\begin{array}{c} D\left(\;\Omega_{S},\Omega_{G}\;\right)=\frac{2\left(\left|\Omega_{S}\cap \Omega_{G}\right|\right)}{\left|\Omega_{S}\right|+\left|\Omega_{G}\right|} \end{array}$$

where *Ω*_*S*_ and *Ω*_*G*_ represent the volumes of segmented result and the ground truth, respectively. DSC is targeted to measure the overlap extent between the segmentation results and ground-truth images, which varies from 0% to 100%. Higher DSC values would be representative of larger overlapping region areas and better segmentation outcomes.

The MCR is defined as follows:

(19) $$\begin{array}{c} M\left(\;\Omega_{S},\Omega_{G}\;\right)=1-\frac{\left|\Omega_{S}\cap \Omega_{G}\right|}{\left|\Omega_{G}\right|}. \end{array}$$

The MCR aims to calculate the proportion of the object region being segmented to the incorrect class, which also ranges from 0% to 100%. In comparison with DSC, the lower value the MCR scores, the fewer regions are being segmented into the mistaken categories and certainly the better segmentation consequence will be obtained.

The MAD is given by

(20) $$\begin{array}{c} d_{M}\left(\;S,G\;\right)=\frac{1}{m_{S}}\overset{m_{S}}{\underset{i=1}{\sum }}\left|\;d_{i}^{SG}\;\right| \end{array}$$

where *S* and *G* denote the boundaries of segmentation results and ground truth, respectively, *m*_*S*_ denotes the total number of pixels that lie on the boundary in the segmentation image, *d*_*i*_^*SG*^ represents the distance from the *i*_*th*_ pixel on the boundary of segmentation result to the nearest pixel on the boundary of ground truth. Therefore, MAD measures the mean absolute boundary distance between segmentation result and ground truth. Obviously, the lower the MAD is, the better the segmentation result is.

The HD is defined as follows:

(21) $$\begin{array}{c} d_{H}\left(\;S,G\;\right)=max\left\{\;\underset{x\in S}{sup}\;\underset{y\in G}{inf}\;d\left(\;x,y\;\right),\underset{y\in G}{sup}\;\underset{x\in S}{inf}\;d\left(\;x,y\;\right)\;\right\}. \end{array}$$

This measurement represents the maximum distance from pixels on the boundary of segmentation result to the closest pixels on the boundary of ground-truth images. In a similar manner, larger HD indicates farther distance between the two boundaries and worse segmentation outcome to be attained.

### 4.3. Segmentation Results

**Figure 3:**
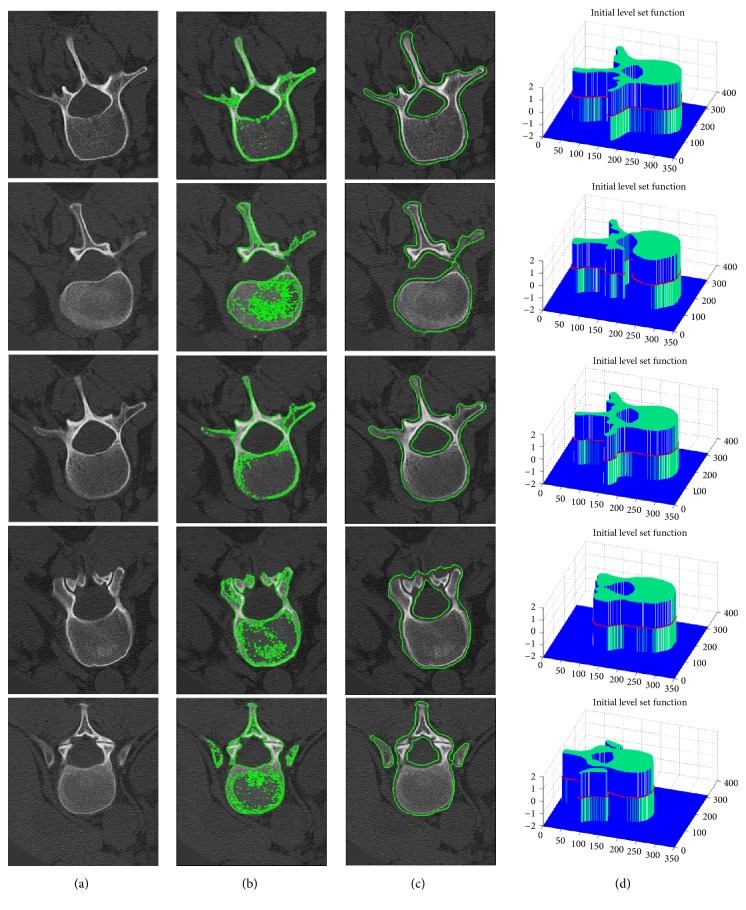
Automatically initialized level set contour of five selected slices. (a) Input images. Initial contours generate by (b) Otsu method, (c) our proposed AILSF. (d) Initialized LSF of our method.

To validate the performance of our approach on CT lumbar vertebrae image segmentation, we have conducted two experiments. The first experiment is designed to evaluate the effect of automatically initialized level set contour by our proposed AILSF, and the corresponding segmentation results are compared with those of Otsu method. As can be seen in Figure 3, the contours automatically initialized by AILSF are, apparently, smoother and more well-defined than the contours initialized via Otsu method. It is able to specifically filter salt-and-pepper noise and make the initialized contour precisely outside and close to the object boundary.

**Figure 4:**
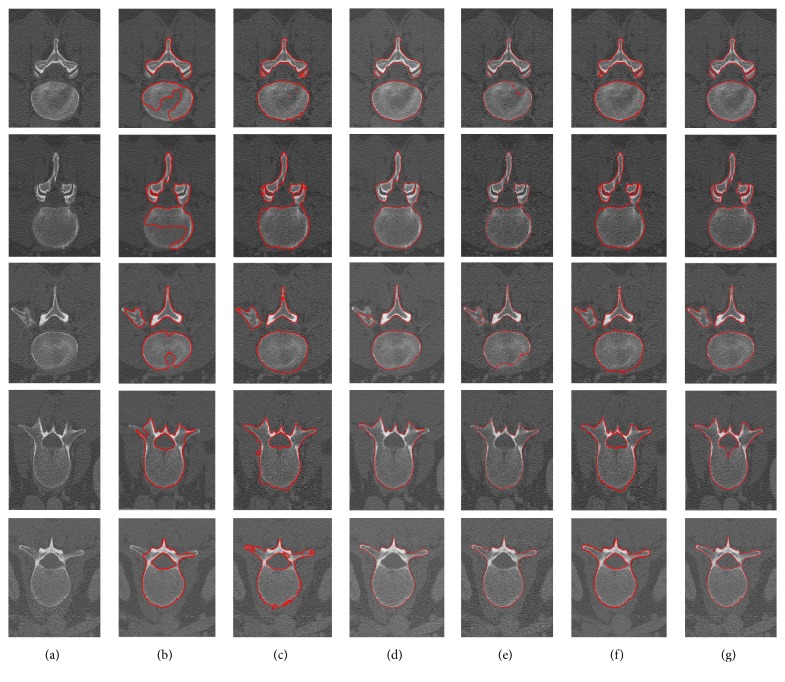
Comparison of the lumbar vertebrae CT image segmentation results with original noise level. (a) Input images, images segmented by (b) the C-V model [15], (c) Lim's model [21], (d) Khadidos' model [30], (e) Liu's model [31], (f) our proposed AGLSA, and (g) ground truth.

**Figure 5:**
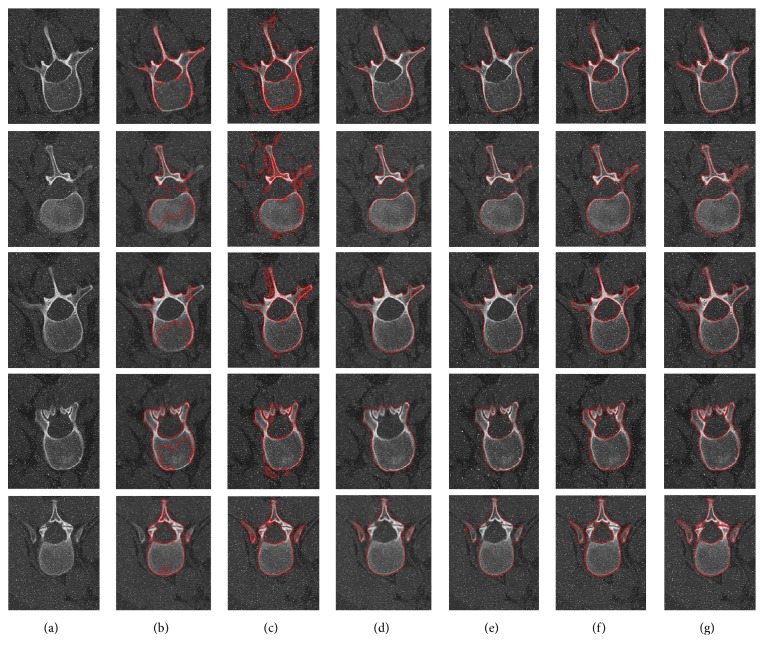
Comparison of the lumbar vertebrae CT image segmentation results added with 3% noise level. (a) Input images, images segmented by (b) the C-V model [15], (c) Lim's model [21], (d) Khadidos' model [30], (e) Liu's model [31], (f) our proposed AGLSA, and (g) ground truth.

**Figure 6:**
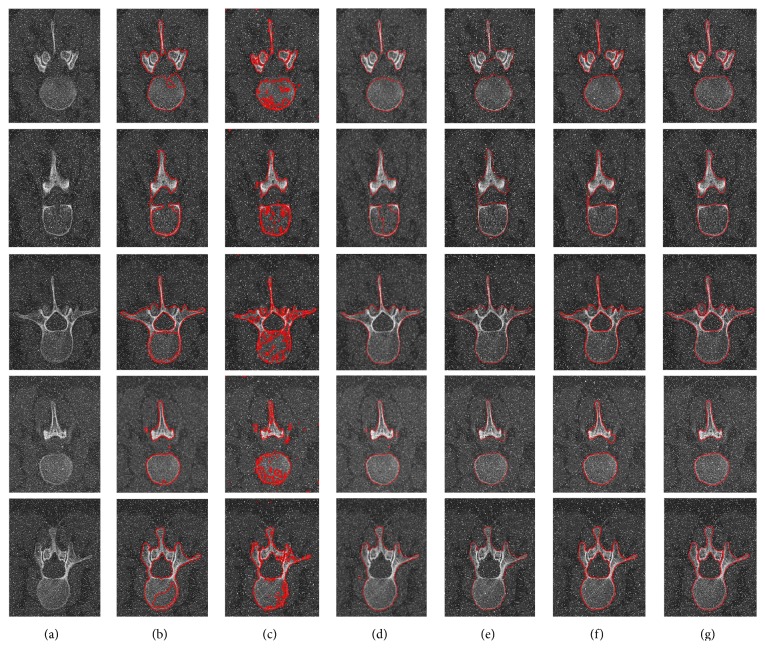
Comparison of the lumbar vertebrae CT image segmentation results added with 7% noise level. (a) Input images, images segmented by (b) the C-V model [15], (c) Lim's model [21], (d) Khadidos' model [30], (e) Liu's model [31], (f) our proposed AGLSA, and (g) ground truth.

The second experiment is performed to evaluate the robustness and accuracy of our proposed AGLSA for lumbar vertebrae CT image segmentation by adding various levels of salt-and-pepper noise. The intuitional comparisons are illustrated in Figures 4, 5, and 6.

Figures 4, 5, and 6 illustrate the comparisons of the segmentation results utilizing the C-V model [15], Lim's model [21], Khadidos' model [30], Liu's model [31], our proposed AGLSA, and ground truth with various levels of salt-and-pepper noise. From these figures, it can be seen that the C-V model performs stable segmentation from different levels of noise, but it is unable to solve the irregular boundary problem and leads to undersegmentation since it assumes that the intensity of the object region is homogeneous. Lim's model is so sensitive to salt-and-pepper noise that it cannot generate continuous contour when the noise level is added to 7%. Moreover, Lim's model raises oversegmentation which is attributed to its edge-based property. Khadidos' model and Liu's model have better segmentation performance than Lim's model because of the utilization of local regional information. However, Khadidos' model and Liu's model failed to segment the inner boundary of the vertebral foramen. It is noticeable that our proposed AGLSA achieves smoother and more accurate segmentation results than others. Furthermore, our proposed AGLSA is robust to various levels of salt-and-pepper noise because of using global region-correlation information.

**Figure 7:**
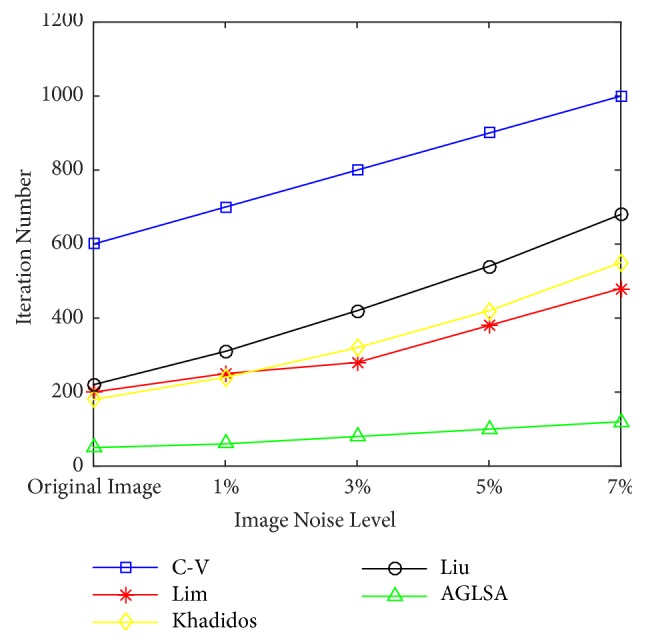
Average iteration number for the segmentation results using the C-V model [15], Lim's model [21], Khadidos' model [30], Liu's model [31], and our proposed AGLSA with different noise levels.

Figure 7 compares the average iteration numbers for level set formulation to achieve convergence via the C-V model [15], Lim's model [21], Khadidos' model [30], Liu's model [31], and our proposed AGLSA with various levels of noise. The *x* − *axes* represent different image noise levels of input image from 0 to 7%. The *y* − *axes* represent the average iteration numbers for level set formulation to achieve convergence. It is obvious that, because of using AILSF to automatically generate a smooth initial contour which is precisely adjacent to the object boundary, our proposed AGLSA has a faster convergence rate and is robust to various levels of salt-and-pepper noise.

Table 1 indicates the quantitative comparison results of our proposed approach with the other four methods. It lists five different criteria: DSC, MCR, MAD, HD, and RT of these methods. It can be seen that Lim's model obtains satisfactory segmentation result under original noise level, but its edge-based property makes it sensitive to the image noise, and the performance under 1%, 3%, 5%, and 7% levels of salt-and-pepper noise is significantly degraded. Although Khadidos' model and Liu's model have better performance, the running times become longer under 7% noise level because the manually initialized contours are sensitive to the image noise. It is remarkable that for images added with 1%, 3%, 5%, and 7% levels of salt-and-pepper noise, our proposed approach outperforms others within all these five criteria since we adopt region correlation of the histograms inside and outside the contour. The conclusion can be drawn that our proposed AGLSA is robust and accurate in presence of salt-and-pepper noise and is capable of converging quickly to the target boundaries.

## 5. Conclusion

In this paper, we present a novel level set formulation for lumbar vertebrae CT image segmentation. Our proposed AGLSA is able to conduct the automatic initialization via AILSF which consists of HMF and GMM, and the corresponding initial contour is smoother and more well-defined than that initialized by Otsu method. Furthermore, a global level set formulation is introduced based on the region correlation which is calculated by Mahalanobis distance of histograms inside and outside the level set contour in order to avoid weak boundary leaking problem. Ultimately, a GVF-based edge-stopping function is utilized to guarantee a fast convergence rate of the level set evolution and to avoid LSF oversegmentation at the same time. Experimental results on clinical images demonstrate that our proposed AGLSA is sufficiently more accurate and robust than the other four models. Moreover, our algorithm performs much more computationally efficient in presence of various levels of salt-and-pepper noise. Our proposed approach could be extended to other applications in medical image segmentation (brain MRI and cardiac ultrasound). The limitation of our approach is that it only copes with the irregular boundaries (double boundary and weak boundary) and image noises in the segmentation. It does not take into account the occlusion of vertebrae caused by the pathological conditions. Shape prior information could be a possible solution to this issue. Further researches and experiments can be conducted to guarantee the robustness and accuracy of segmentation results with pathological conditions, such as tumors, lesions, and implanting pedicle screws.

## Figures and Tables

**Table 1:** Average DSC (%), MCR(%), MAD (pixel), HD (pixel), and RT (second) with standard deviation of various segmentation results with different levels of noise.

Input images	Criteria	C-V [15]	Lim [21]	Khadidos [30]	Liu [31]	AGLSA
Original images	DSC(%)	68.53 ± 3.06	89.01 ± 1.21	87.26 ± 3.25	86.67 ± 2.48	92.08 ± 2.37
	MCR(%)	14.85 ± 7.52	8.53 ± 4.34	8.65 ± 2.27	8.92 ± 3.06	5.23 ± 2.06
	MAD(P)	15.66 ± 8.64	9.47 ± 4.65	10.15 ± 3.34	11.90 ± 2.43	6.94 ± 4.01
	HD (P)	26.17 ± 3.64	15.62 ± 2.40	15.70 ± 1.87	18.40 ± 3.18	11.54 ± 3.45
	RT (S)	42.3 ± 1.6	12.5 ± 2.1	16.7 ± 3.2	20.3 ± 1.5	5.9 ± 1.5

Images with 1% salt and pepper noise	DSC(%)	63.26 ± 5.86	81.74 ± 4.52	83.85 ± 2.88	84.53 ± 3.05	86.01 ± 3.23
	MCR(%)	21.55 ± 3.70	13.06 ± 3.14	11.67 ± 2.10	9.17 ± 2.76	8.93 ± 2.31
	MAD(P)	30.23 ± 7.41	19.94 ± 2.86	17.56 ± 4.81	15.90 ± 3.15	14.67 ± 3.74
	HD (P)	51.14 ± 5.35	25.81 ± 4.17	22.68 ± 3.11	20.36 ± 4.73	19.75 ± 1.50
	RT (S)	50.5 ± 2.6	20.3 ± 2.6	26.5 ± 1.7	30.1 ± 2.3	8.5 ± 2.0

Images with 3% salt and pepper noise	DSC(%)	56.30 ± 2.04	70.55 ± 2.63	79.13 ± 2.43	80.47 ± 1.56	82.12 ± 3.22
	MCR(%)	29.15 ± 5.33	27.21 ± 3.58	18.26 ± 2.58	17.62 ± 3.24	14.34 ± 2.06
	MAD(P)	40.26 ± 4.60	25.93 ± 5.11	24.78 ± 1.09	23.13 ± 2.54	19.50 ± 1.86
	HD (P)	69.08 ± 5.68	35.60 ± 3.76	29.31 ± 4.72	25.94 ± 3.69	23.65 ± 4.33
	RT (S)	70.8 ± 4.5	26.7 ± 1.8	36.3 ± 2.6	42.6 ± 1.8	13.0 ± 1.7

Images with 5% salt and pepper noise	DSC(%)	50.23 ± 1.12	65.31 ± 3.08	73.01 ± 3.51	75.54 ± 3.42	78.65 ± 2.89
	MCR(%)	33.40 ± 4.67	31.70 ± 2.36	27.94 ± 3.62	26.73 ± 2.50	20.86 ± 4.57
	MAD(P)	45.15 ± 3.50	28.67 ± 1.45	28.75 ± 2.45	25.65 ± 3.26	23.01 ± 3.15
	HD (P)	70.78 ± 5.37	39.45 ± 5.27	33.50 ± 4.21	31.32 ± 3.18	25.94 ± 2.78
	RT (S)	81.3 ± 2.4	45.8 ± 3.1	49.1 ± 2.3	56.8 ± 2.4	16.3 ± 2.5

Images with 7% salt and pepper noise	DSC(%)	47.86 ± 6.03	58.93 ± 5.64	70.49 ± 3.01	72.12 ± 2.65	75.40 ± 3.32
	MCR(%)	40.11 ± 3.27	38.65 ± 3.77	32.71 ± 3.57	31.63 ± 3.72	28.23 ± 4.07
	MAD(P)	48.67 ± 7.10	31.51 ± 4.06	30.62 ± 3.26	28.16 ± 3.44	25.65 ± 2.28
	HD (P)	74.55 ± 5.89	43.25 ± 4.15	39.78 ± 4.80	37.25 ± 2.15	29.85 ± 3.48
	RT (S)	89.4 ± 2.1	60.5 ± 2.3	65.7 ± 1.2	70.8 ± 2.4	19.1 ± 1.6

## Data Availability

The data used to support the findings of this study are available from the corresponding author upon request.
